# Salivary Gland Anlage Tumor: A Case Report on Abnormal Breathing Found in a Late-Preterm Infant

**DOI:** 10.7759/cureus.64921

**Published:** 2024-07-19

**Authors:** Austin Han, Carter Bruett, Kristen Thomas, Zeyar Htun, Zahrah Taufique

**Affiliations:** 1 Division of Neonatology, Department of Pediatrics, NYU Langone Hospital - Long Island, Mineola, USA; 2 Department of Pathology, NYU Langone Health, New York, USA; 3 Division of Neonatology, Department of Pediatrics, NYU Langone Hospital – Long Island, Mineola, USA; 4 Division of Pediatric Otolaryngology, Department of Otolaryngology - Head and Neck Surgery, NYU Langone Health, New York, USA

**Keywords:** preterm-born infant, infant, neonatal respiratory distress syndrome, salivary gland neoplasms, nasopharyngeal neoplasms, salivary gland anlange tumor

## Abstract

There are many etiologies for respiratory distress in newborns, one of the rare causes being nasopharyngeal tumors. Of that category, salivary gland anlage tumor (SGAT) is exceedingly rare. Symptoms of SGAT vary by patient, but the most common presenting symptom is respiratory distress. The rarity of SGAT and infantile nasopharyngeal tumors in general can lead to delayed diagnosis in newborns with respiratory distress. We report an unexpected and incidental finding of this potentially life-threatening condition in the neonatal population. A preterm male infant with respiratory distress, who was undergoing a neurological workup for new hypotonia, was found to have an incidental nasopharyngeal mass after brain MRI. Upon eventual minimally invasive endoscopic surgical excision and pathologic workup for the mass, the patient was diagnosed with SGAT. The patient has since been with outpatient follow-up visits with no evidence of recurrence of the mass. The purpose of this report is to present a rare and often overlooked life-threatening diagnosis of respiratory distress in the neonatal population.

## Introduction

Congenital nasopharyngeal masses are one of the etiologies to consider in a newborn with respiratory distress. However, they are a rare cause since congenital head and neck anomalies have a 5.5% prevalence among all congenital anomalies [1]. Symptoms usually vary, with feeding difficulties and acute respiratory distress being the most common and usually present within the first 24 hours of life. Because newborns are obligate nasal breathers, these masses can lead to increasing respiratory distress due to the obstruction and in some cases may require emergent airway access [2]. These tumors can be benign or malignant with teratomas being the most common [1].

Salivary gland anlage tumors (SGATs), formerly known as congenital pleomorphic adenoma, is a specific rare benign tumor of the nasopharynx. According to the 2017 World Health Organization Classification of Head and Neck Tumors, SGAT is one of the three types of epithelial tumor that is unique to the nasopharynx, the others being malignant salivary gland tumors and nasopharyngeal papillary adenocarcinoma [3]. These masses are usually identified through imaging scans and through flexible fiberoptic laryngoscopy. Due to the rarity of these masses in neonates, the index of suspicion for nasopharyngeal tumors in infants with respiratory distress is usually low.

There is some debate regarding the neoplasticity of SGAT. Since it was first described in 1980, SGAT has held alternative monikers such as “squamous cell proliferative lesion” and “congenital pleomorphic adenoma,” although at this point neither is appropriate for diagnostic use [4,5]. These historical terms evoked a neoplastic process, although it was debated from the very first cases. The consistent midline presentation and indolent course supported a benign neoplastic etiology in the eyes of most early authors. Recently published cases of SGAT have explored this question with genetic testing and have found no significant driving mutations in SGAT [6,7]. This lends support to the assertion that SGAT is more likely a hamartomatous process. Regardless, this lesion can be life-threatening. Infants are obligatory nasal breathers and the nasal congestion associated with this lesion must be addressed expeditiously, which requires awareness of this entity.

There are 42 previously reported cases of SGAT, with this report marking the 43rd published case [5]. Several review articles to date have addressed the clinical features and management of SGAT [6,7]. The histopathology of SGAT has been discussed across many of the articles, although no critical scoping review is apparent. SGAT resembles the developing salivary gland and histologically reveals a mix of salivary epithelial and myoepithelial cells. There are also varying findings of mitotic activity ranging from absent to numerous. It has also been associated with a Ki-67 proliferation index ranging from 1 to 30 percent. The present report introduces a case of SGAT with an unusual morphology and elevated proliferation index, which expands the histopathologic spectrum associated with SGAT. In doing so, we raise awareness of this important diagnosis to consider in this clinical context.

## Case presentation

Clinical presentation and diagnostic workup

A late preterm 34 2/7 weeks gestation male infant Twin A was born to a 38-year-old G5P4004 mother via cesarean section secondary to preterm labor and breech positioning. Pregnancy was complicated by dichorionic diamniotic twin pregnancy and breech positioning. Prenatal testing was all negative except for an unknown group B *Streptococcus* (GBS) status. The mother received betamethasone once three hours prior to delivery. At delivery, there was an artificial rupture of the membrane with clear fluid. APGARs were 8 and 9 at the one minute and five minutes, respectively. The infant was admitted to the neonatal intensive care unit (NICU) in room air for prematurity.

On initial presentation to the NICU, the patient was found to be desaturating on room air with an oxygen saturation of 82% on pulse oximetry. He had associated symptoms of intermittent grunting, subcostal retractions, and diminished breath sounds. He was initially started on respiratory support of bubble CPAP+6. The initial blood gas was within normal limits, and the chest X-ray was significant for respiratory distress syndrome. However, due to respiratory distress, the oxygen was steadily increased to a maximum setting of non-invasive mechanical ventilation (NIMV) rate of 30 pressures of 20/6. Ultimately, the patient was weaned to room air on day of life 6.

**Figure 1:**
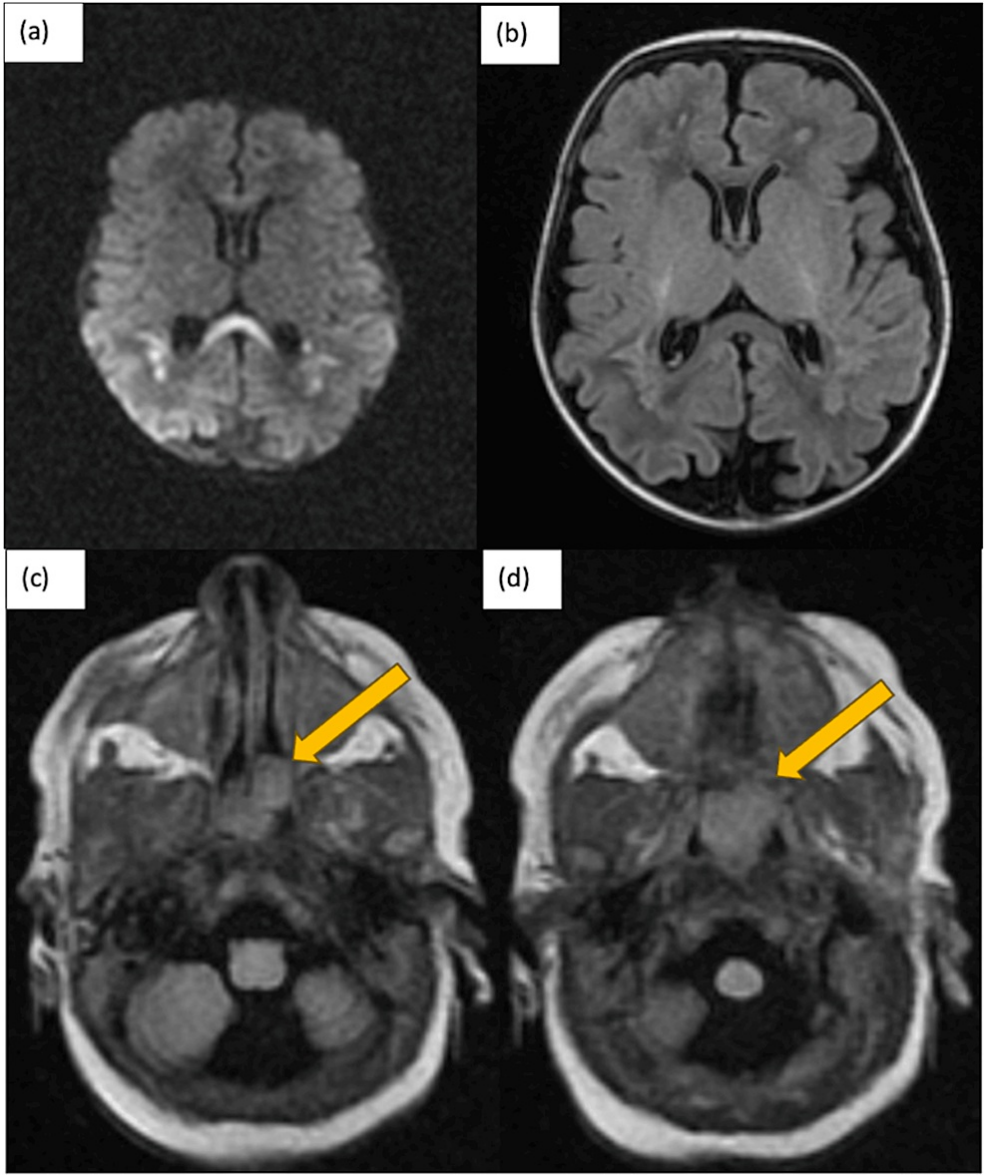
Initial brain MRI done on day of life 10. (a, b) Neurologic radiographic findings of the diffusion abnormality of the corpus callosum and posterior periventricular white matter compatible with HIE. Additional patchy T1/T2 signal abnormality of the bilateral deep white matter without corresponding diffusion abnormality likely periventricular leukomalacia (PVL)/hypoxic ischemic encephalopathy (HIE). (c, d) Findings of the nonspecific polypoid soft tissue partially filling the nasopharynx with no definite evidence of osseous choanal atresia.

On day of life 8, the patient was seen to have a new decreased tone and head lag with the development of pursed lip breathing. The head ultrasound was unremarkable. Brain MRI was done on day of life 10, which showed a diffusion abnormality of the corpus callosum and posterior periventricular white matter compatible with hypoxic-ischemic encephalopathy (HIE) (Figure 1a-1b). There was also decreased myelination of the corticospinal tract for age. A magnetic resonance angiography (MRA)/magnetic resonance venography (MRV) was subsequently done, which confirmed no large vessel occlusion or severe stenosis. The patient was followed by the pediatric neurology team, had normal electroencephalogram (EEG) testing and brainstem auditory evoked response (BAER) testing, and was cleared for outpatient follow-up.

**Figure 2:**
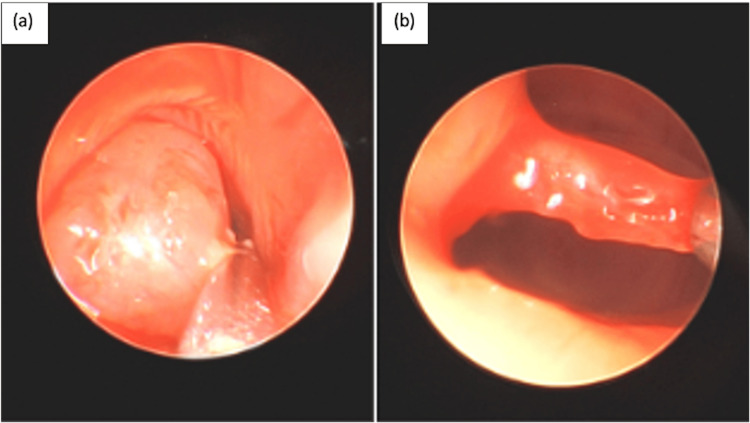
Intra-operative imaging on day of life 14. (a) Soft polypoid mass causing ball-valving obstruction of the nasal pathway. (b) A very thin stalk attaches the mass to the posterior septum.

**Figure 3:**
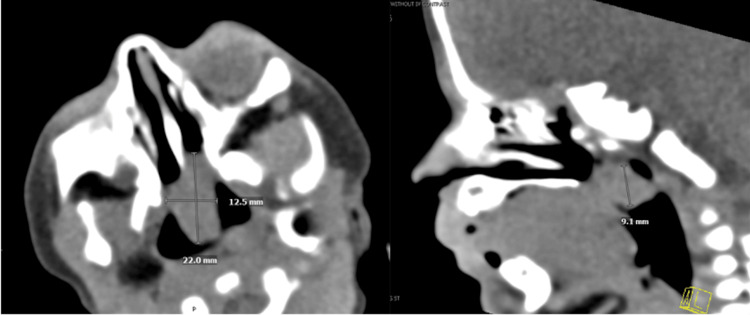
CT facial bone done on day of life 12. Depicts the dimensions of the mass. Nonspecific 2.2 cm polypoid soft tissue inseparable from the soft palate with extension into the nasopharynx, oropharynx, and bilateral coanae. No associated bone destruction.

**Figure 4:**
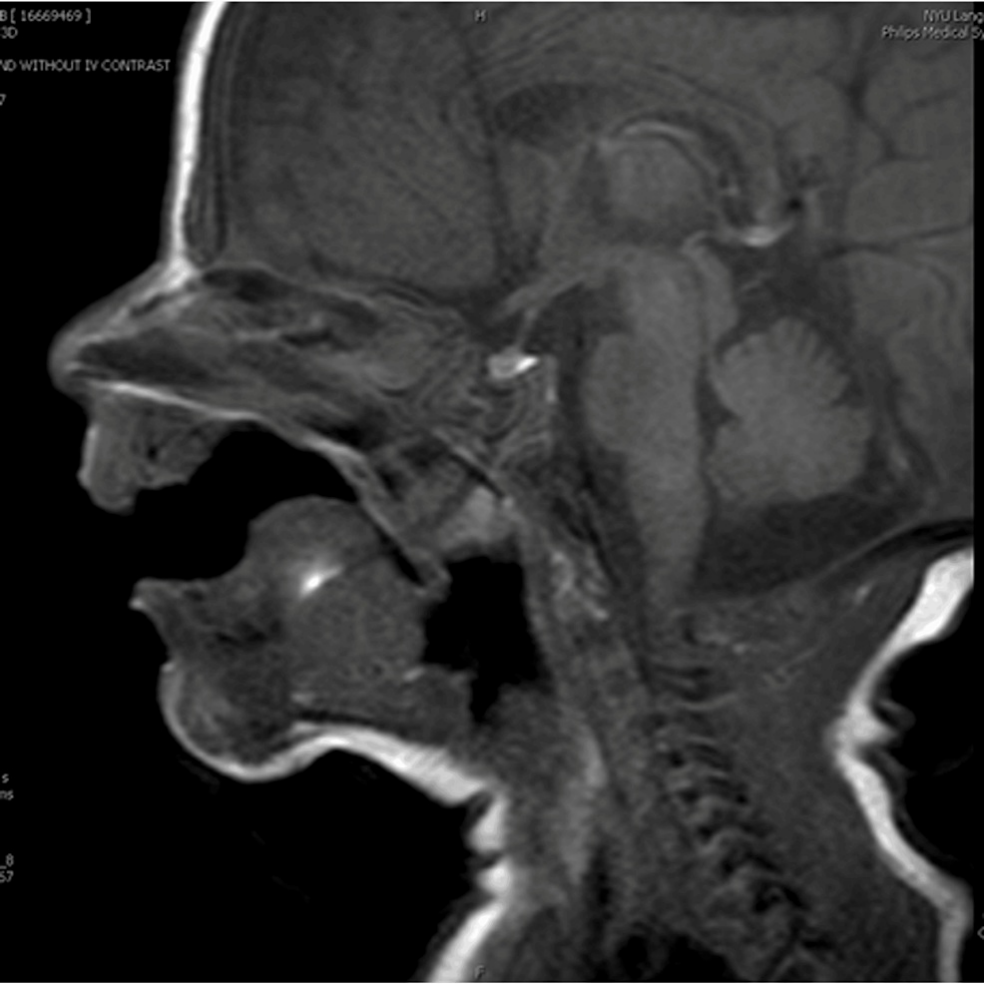
MRI face with contrast done on day of life 13 revealed similar findings of the mass with no evidence of vascularization.

The initial brain MRI also showed an incidental finding of a non-specific polypoid soft tissue partially filling the nasopharynx (Figure 1c-1d), which probably contributed to the pursed lip breathing exhibited by the infant. Therefore, ENT was consulted. A flexible fiberoptic laryngoscopy was done through bilateral nasal cavities. A soft tissue mass ball-valving into the posterior nasal cavity was seen with an intact remainder of the upper airway (Figure 2a). A CT scan of the facial bone was performed on day of life 12, which showed a non-specific 2.2 cm polypoid soft tissue inseparable from the soft palate with extension into the nasopharynx, oropharynx, and bilateral choanae (Figure 3). A face MRI with contrast was done on day of life 13, which confirmed a mass with no vascularization (Figure 4).

Treatment objectives

The clinical findings were discussed with the parents of the patient. The next step of care was established to undergo an excision of the nasopharyngeal mass under general anesthesia.

The main objective was to remove the nasopharyngeal mass that could have caused the respiratory distress seen in this patient and prevent future cases of airway compromise. The mass was sent to pathology for histologic diagnosis and to plan for additional imaging or tests if necessary. The agreed surgical procedure was a minimally invasive endoscopic excision. Typically, the surgery is well tolerated with the potential for same-day discharge if done through outpatient circumstances. However, imaging should be done prior to surgical intervention given the unknown nature of the mass to rule out vascular connections and risk for bleeding.

Treatment progress

On day of life 14, the patient had an uncomplicated endoscopic excision of the nasopharyngeal mass under general anesthesia. During the procedure, the mass was found to be attached to the posterior septum by a very thin stalk (Figure 2b). A 3-0 suture was used to retract the soft palate as the mass would be too large to deliver through the nose in preparation for delivery through the mouth. The stalk was ligated and the mass was pushed down through the oral cavity and removed through the mouth. The fibrous stalk was then removed with cup forceps and sent for biopsy at the margin. Ultimately, the lesion measured 2.5 x 1.5 cm. It was exophytic, homogenous, and fleshy colored and also sent for biopsy. The patient was then discharged from the NICU on day of life 19.

The patient has had continued outpatient follow-up visits with ENT. A repeat flexible fiberoptic laryngoscopy was done in the office. He had clear bilateral nasal passageways with no visible regrowth of the mass to date, with the last follow-up visit at four months of age.

Results

**Figure 5:**
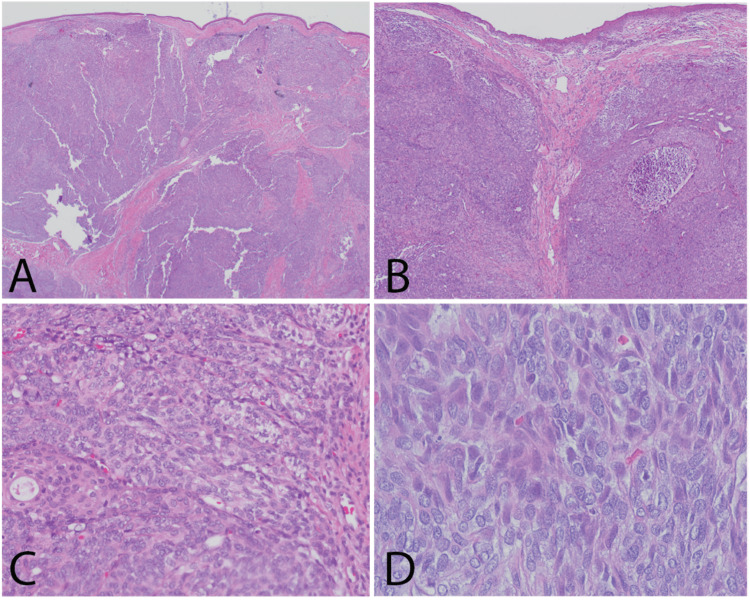
H&E stained sections of the tumor. (a) Cellular lobules of the tumor are surfaced by a thin layer of stratified squamous epithelium and bounded by hypocellular fibrous stroma. (b) Tumor lobules elevate the surface mucosa and contain a variable population of epithelial and myoepithelial cells and occasional duct formation. (c) The tumor cells are arranged in nodules and contain occasional ducts with a squamous phenotype and focal cells with clear cytoplasm. (d) The tumor cells are biphenotypic with one population exhibiting angular nuclei and fine chromatin with an eosinophilic cytoplasm and the other demonstrating ovoid nuclei with coarse chromatin and amphophilic to clear cytoplasm.

The tissue was fixed in formalin, embedded in paraffin, sliced into standard 4-micron sections, and prepared with hematoxylin and eosin stain. An initial review of the pathologic specimen showed nonkeratinizing stratified squamous mucosa with a cellular submucosal proliferation composed of multiple solid nodules separated by a relatively hypocellular stroma and a network of delicate linear and branching small duct-like and glandular structures and nests of squamous epithelium within a variably fibromyxoid stroma (Figure 5a-5b). The cellular epithelial nodules had a somewhat basaloid appearance, composed of nests and intersecting trabeculae with poorly formed peripheral tubules. The cells were relatively large and had round to oval nuclei, inconspicuous nucleoli, and abundant eosinophilic cytoplasm (Figure 5c). There was no evidence of overt cellular pleomorphism or atypia, but frequent mitotic and apoptotic figures could be identified (Figure 5d).

**Figure 6:**
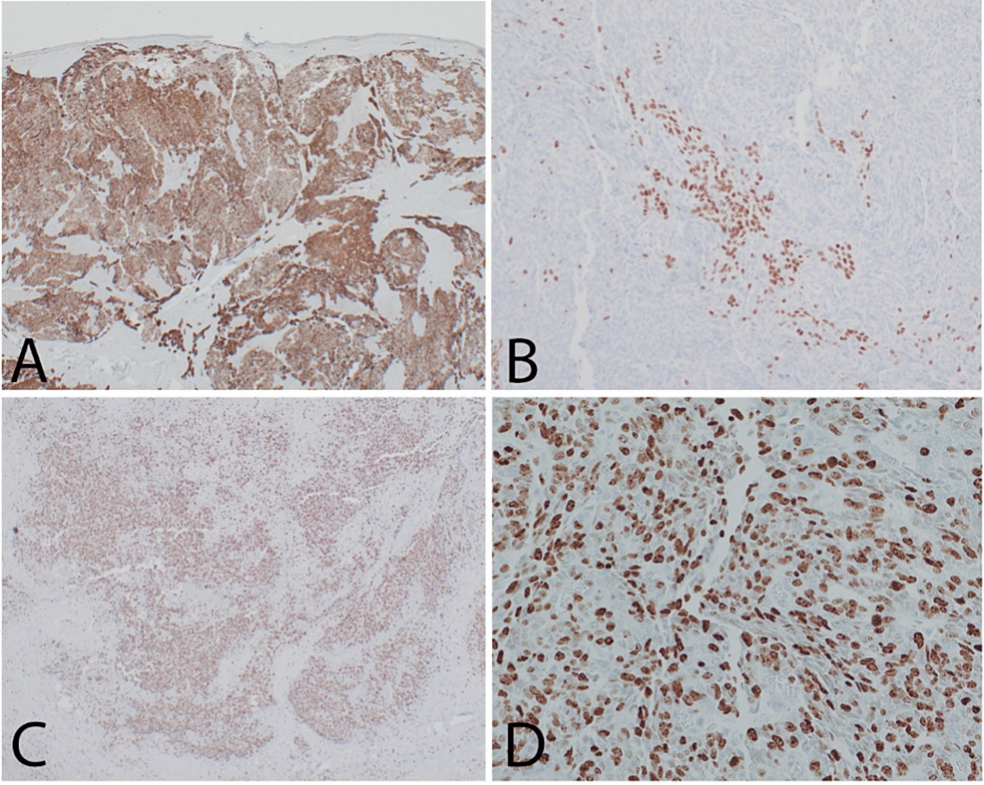
Immunohistochemical stains. (a) CAM5.2 demonstrates the epithelial phenotype of the tumor. (b) p40 highlights localized areas with a salivary-type myoepithelial population. (c) Ki-67 highlights a significant percentage of tumor cells, suggesting an atypical propensity for growth. (d) Ki-67 demonstrates a 75% positivity rate in some tumor nodules.

Given the unusual histomorphology, a panel of immunohistochemical stains was utilized to narrow the differential diagnosis. CAM 5.2, CK7, INI-1, p63, p40, SMA, Ki-67, HMB-45, GFAP, S-100 protein, beta-catenin, SOX-10, CD117, desmin, p53, and synaptophysin were all completed at an internal hospital laboratory with validated controls. The results supported a biphasic epithelial and myoepithelial salivary-type etiology. The epithelial component of the tumor was diffusely and strongly positive for cytokeratins CAM 5.2 and CK7 (Figure 6a). The myoepithelial components and epithelial basal cells were highlighted by SMA and both p63 and p40 (Figure 6b). INI-1 was retained, and the cells had a diffuse membranous and cytoplasmic staining pattern with beta-catenin with no nuclear staining. The Ki-67 was focally high, up to 75% in some tumor nodules (Figure 6c-6d). The tumor cells were negative for HMB-45, GFAP, S-100 protein, SOX-10, CD117, desmin, and synaptophysin and expressed wild-type p53. Combining the clinical findings, histomorphology, and immunohistochemical findings, the diagnosis of salivary gland anlage tumor was rendered.

## Discussion

Congenital nasopharyngeal tumors are rarely seen, and there are still no clear patient management guidelines available. Embryologically, the nasopharynx is formed from the intersection of the neural axis with the alimentary and respiratory tracts. Tumors of this area bear histological similarities to these cell lineages. Although diagnosis is mainly accomplished antenatally, there are some nonspecific prenatal findings that may be related to congenital nasopharyngeal tumors. A study revealed that about 20% of neonates presented with polyhydramnios or elevated alpha-fetoprotein [1]. When the spectrum of congenital nasopharyngeal tumors is considered, SGAT stands out as one of the three epithelial tumors unique to the nasopharynx. The etiology of SGAT remains unknown, with recent genetic studies failing to reveal any molecular drivers of this disease, thus supporting the theory that SGAT is a hamartomatous process, rather than a neoplastic one [7].

The symptoms that are associated with SGAT vary with patients and usually present early in life. However, in a retrospective study of 42 case reports, it was found that 30/42 experienced respiratory distress, and 13/42 experienced upper airway obstruction [5]. The disease is male-dominant, with 74% of cases reported in male infants (a 3:1 male-to-female ratio). SGAT is consistently located within the nasopharynx, often attached to the posterior nasal septum and extending into the nasal cavity. Interestingly, in six patients, there has been spontaneous expulsion of the tumor [2,7-10]. The spontaneous dislodgement may be attributed to the finding of a thin stalk at the site of attachment, as seen in our patient, or similar pedunculation in other reported cases. Some cases have reported a sessile mass instead of a pedunculated one, and in one case, the lesion was noted to occlude the esophagus rather than the nasal cavity [11]. The mainstay treatment for these tumors is surgical resection. A study reported that 36/42 patients had surgical resection of the tumor [5]. The prognosis after resection is very good. To date, there have been no reported cases with recurrence [12].

When diagnosing SGAT, imaging is key to understanding the size of the mass and defining its characteristics. CT and/or MRI can be used to obtain the characteristics and also understand vasculature regarding the mass [13]. A flexible fiberoptic nasal endoscopy can be used to view the mass grossly and also for planned excision of the mass.

The first case of SGAT was described in 1994 in a case series of nine patients. Since then, 43 cases have been published (including the present case). SGAT has a particular predilection for the midline and is often attached by a thin stalk or pedicle to either the nasal septum or posterior pharyngeal wall [2]. Histologically, SGAT resembles the developing salivary gland, which was the source of the historical term “congenital pleomorphic adenoma” [8]. This is evidenced by the biphasic nature of the tumor, exhibiting a mix of salivary epithelial and myoepithelial cells. Reports of mitotic activity vary from absent to numerous, although the finding of infrequent mitoses is more common. Ki-67 proliferation index reports from previous publications suggest a range from 1 to 30 percent. Necrosis has been reported, either focally or extensively, and has been attributed to the potential for torsion and devascularization given the thin stalk that often attaches the mass to the nasopharynx.

In the current case, we present findings that expand the spectrum of tumor morphology for SGAT to include a heightened proliferation index and more cellular appearance. The nodules of the solid tumor in our case appeared large and dominant, pushing up against the surface epithelium and occupying the bulk of the tumor, similar to a case reported by Peters et al. [7]. This is not described in other cases. Our case features only rare squamous islands with no cyst formation. Mitotic figures were mostly rare in our case, with some areas exhibiting up to two mitoses per square millimeter. The proliferation index as measured by Ki-67 in our case was up to 75% in areas of the tumor. The clinical features were classic for SGAT, the histology was clearly supportive of the diagnosis, but the unexpected combination of features raised concern about the aggressiveness of this tumor. To date, no recurrence has been detected in this case, which remains consistent with the diagnosis of SGAT.

## Conclusions

SGAT is a very rare type of the already uncommon congenital nasopharyngeal group of tumors. It is one of the many etiologies of respiratory distress in newborns and should be included in the differential diagnosis for these situations. SGAT can be diagnosed from its unique histomorphology, although immunohistochemistry and genetic testing can help the pathologist arrive at the diagnosis. The evidence supports that SGAT is a hamartomatous lesion of developing salivary gland tissue. We have presented a case that has more worrisome histologic features that may raise the possibility of a more aggressive variant. Awareness of this broadened spectrum, and the still indolent outcome of this case, should improve recognition. There is still a lack of a formal guideline regarding management in hospitals and during out-patient follow-ups. Although very rare overall, SGAT has an excellent prognosis with surgical resection. Given the rarity of this pathology, there are still unclear guidelines, and further research could be done to study this mass and fully understand its components.
